# Evaluation of blood and bone marrow in selected canine vector-borne diseases

**DOI:** 10.1186/s13071-014-0534-2

**Published:** 2014-12-02

**Authors:** Anna S De Tommasi, Domenico Otranto, Tommaso Furlanello, Silvia Tasca, Cinzia Cantacessi, Edward B Breitschwerdt, Dorothee Stanneck, Filipe Dantas-Torres, Gad Baneth, Gioia Capelli, Donato de Caprariis

**Affiliations:** Dipartimento di Medicina Veterinaria, Università degli Studi di Bari, Valenzano, Bari Italy; Laboratorio d’Analisi Veterinarie “San Marco”, Padova, Italy; Department of Veterinary Medicine, University of Cambridge, Cambridge, UK; Department of Clinical Sciences, Intracellular Pathogens Research Laboratory, Center for Comparative Medicine and Translational Research, College of Veterinary Medicine, North Carolina State University, 1060 William Moore Drive, Raleigh, NC 27607 USA; Department of Immunology, Aggeu Magalãhes Research Institute, Oswaldo Cruz Foundation, Recife, Pernambuco Brazil; School of Veterinary Medicine, Hebrew University, Rehovot, Israel; Istituto Zooprofilattico Sperimentale delle Venezie, Laboratory of Parasitology, Legnaro, Italy; Bayer Animal Health GmbH, Leverkusen, Germany

**Keywords:** Bone marrow, Cytology, Vector-borne pathogens, Dysplasia, Secondary dysmyelopoiesis

## Abstract

**Background:**

Bone marrow (BM) is a major hematopoietic organ that can harbour a variety of vector-borne pathogens; however, knowledge of BM pathological changes in dogs infected with vector-borne pathogens is limited. Thus, the aim of the present study was to assess the pathological changes in canine BM associated with natural infections by four vector-borne pathogens, as well as to determine the relationships between such changes and abnormalities of the peripheral blood.

**Methods:**

Cytological disorders and pathological changes of the BM of 83 dogs naturally-infected with one or more of four vector-borne pathogens (i.e., *Anaplasma platys*, *Leishmania infantum*, *Babesia vogeli* and *Hepatozoon canis*) were evaluated and compared with the corresponding hematological findings.

**Results:**

Dysgranulopoiesis and dysmegakaryocytopoiesis were the most frequently observed BM abnormalities in infected dogs. Erythroid suppression, and lymphocytic, monocytic and macrophage hyperplasia were also observed. Interestingly, associations between suppression and hyperplasia of specific cell lines in the marrow and corresponding changes in numbers of circulating peripheral blood cells were not observed.

**Conclusions:**

Infections with one or more of the vector-borne pathogens examined in this study should be considered as differential diagnoses for secondary dysmyelopoiesis.

## Background

The bone marrow (BM) is the major hematopoietic organ and a primary lymphoid tissue. In dogs, the BM can become infected with pathogens such as *Leishmania infantum* [1], *Hepatozoon canis* [2] and *Anaplasma platys* [3]; therefore, the BM is considered a sensitive tissue for the detection of these pathogens [4,5]. Since the above-mentioned pathogens are transmitted by arthropod vectors, they are commonly referred to as canine vector-borne diseases (CVBDs); CVBDs are widely distributed and highly prevalent throughout much of the world, including the southern Mediterranean region [6]. Furthermore, multiple canine vector-borne pathogens can simultaneously infect the same host, thereby causing co-infections, which may exacerbate disease severity and alter clinical and pathological scenarios associated with single infections, thus complicating diagnosis, treatment and prognosis in co-infected dogs [7]. The presence of vector-borne pathogens in the BM may induce substantial alterations in erythrocyte, granulocyte, monocyte, lymphocyte and thrombocyte numbers and/or function. Despite the high prevalence of CVBDs [6], only a few studies have investigated BM alterations in dogs infected solely by *L. infantum* [8-10] or by *Ehrlichia canis* [11]. In these studies, BM alterations associated with infections by *L. infantum* included emperipolesis, megakaryocytes dysplasia and BM aplasia, whereas myelosuppression was induced by *E. canis* infection. However, thus far, knowledge of the BM alterations associated with infections by other CVBDs, including *H. canis* and *B. vogeli*, as well as with co-infections by multiple CVBD pathogens, is lacking. Therefore, the aim of the present study was to provide insights into the BM changes that occur in dogs naturally-infected by one or more of four vector-borne pathogens (i.e. *A. platys, B. vogeli, H. canis* and *L. infantum*) and to determine the relationships between such changes and corresponding peripheral blood abnormalities.

## Methods

### Ethics statement

Animals included in this study were handled according to the principles of Good Clinical Practice (VICH GL9 GCP, 2000). The study design and the experimental procedures were approved and authorized by the Italian Ministry of Health (authorization number DGSA nu. 0001997; 04/02/2011; cf. [12,13]).

### Animals

Samples from 83 crossbred dogs, 47 males and 36 females, of different age (ranging from 3 months to 6 years of age), unequivocally diagnosed with one or more CVBD (based on serological, cytological and molecular assays performed on a variety of tissues) were selected from a collection of samples examined in previously published studies [12,13]. Dogs were selected based on the following criteria: (1) positivity to one or more vector-borne pathogens documented by cytology and molecular assays and (2) the suitability of blood and BM smears for subsequent cytological evaluation. BM smears with evidence of blood contamination (i.e. high number of mature neutrophils, numerous platelet aggregates and few marrow cells) were excluded from the study. The selected cases were divided into 4 groups according to the detected pathogen(s) (which included *A. platys*, *B. vogeli, H. canis*, and *L. infantum*, Table 1). No dog included in this study was *E. canis* sero-reactive or PCR positive.

**Table 1:** **Group classification of 83 dogs according to the infecting vector-borne pathogens**

**Group**	**CVBD**		**N° of dogs**
**Ap**	*Anaplasma platys*		10
**Hc**	*Hepatozoon canis*		33
**Li**	*Leishmania infantum*		13
**Co-i**	Co-infections	*H. canis - L. infantum*	12
		*H. canis - A. platys*	10
		*H. canis – Babesia vogeli*	2
		*A. platys -Babesia vogeli*	1
		*H. canis - A. platys - Babesia vogeli*	2
**Tot**			83

### Sample collection and laboratory procedures

Blood samples were collected from the brachial or jugular vein of each dog and a complete blood count (CBC) was performed using an automatic cell counter (Abbott Cell-Dyn 3700, Laboratorio di Analisi Cliniche Veterinarie, ACV Triggiano, Bari). Blood and buffy coat smears were prepared and stained with the MGG Quick Stain (Bio Optica Spa, Milan, Italy). Bone marrow aspirates were obtained under local anesthesia with lidocaine hydrochloride 2%, (Lidocaina 20, Pfizer Olot, S.L.U., Spain; 1–2 ml SC). In particular, BM specimens were collected from the iliac crest using Rosenthal needles (16 or 18 gauges) and 10 ml syringes and Giemsa-stained prior to microscopical examination to evaluate cytological changes.

### Cytological examination

A 500-cell differential count was performed on BM smears; the myeloid to erythroid (M:E) ratio, myeloid maturation index (MMI), erythroid maturation index (EMI) were calculated. The maturation index was defined as the ratio between the number of proliferative phase cells and the number of maturation phase cells in the BM (typically 1:4 in mammals [14]); the MMI is defined as (myeloblasts + promyelocytes + myelocytes) ÷ (metamyelocytes + bands + segmenters), while the EMI is defined as (rubriblasts + prorubricytes) ÷ (rubricytes + metarubricytes) [15]. Reference ranges used in this study for each cell type and for the different ratios have been reported elsewhere [16,17].

The mean number of megakaryocytes was determined for each dog at low magnification (i.e. 10x); megakaryocytic hyperplasia was defined as >3 cells/low-power field [18]. The percentages of myeloid precursors, erythroid cells, and megakaryocytes with dysplastic features were determined. Dysplasia was defined as the occurrence of dysplastic features in over 10% of the counted cells [19,20]. The term ‘depletion’ of one or more BM cell lineages was used for dogs with peripheral cytopenia and BM within reference intervals or hypoplastic BM with a normal or reduced maturation index. Conversely, ‘suppression’ was used to indicate dogs with peripheral cytopenia, accompanied by BM hypoplasia and an increased maturation index [21].

### Statistical analysis

The Fisher’s exact test was used to evaluate the differences in the percentages of abnormalities among dogs with a single infection and between dogs with single infection and those with co-infection. Data were organized in several 2X2 contingency tables comparing, for each abnormality, dogs with a specific vector-borne disease with dogs with another vector-borne disease or a co-infection status, with the aim to reveal a significant higher prevalence of a given abnormality in the two groups. A publicly available online software was used (©2014 GraphPad Software, Inc. at: http://www.graphpad.com/quickcalcs/contingency2/).

## Results

**Figure 1:**
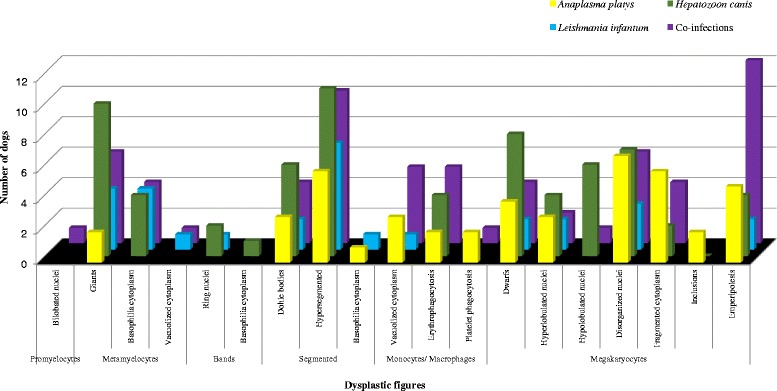
**Frequency of dysplastic figures of the bone marrow of 83 dogs naturally infected by different CVBD pathogens.**

CBC and BM findings are summarized in Tables 2, 3 and 4, respectively; the main dysplastic figures observed in the BM samples are reported in Table 4 and Figure 1. Most BM specimens did not display alterations in cellularity; moreover, the blast cell count was <6% in all dogs, therefore the occurrence of acute myeloproliferative disorders, as a cytopathological differential diagnosis, was excluded. Out of 83 dogs included in this study, only 5 (6%) had CBC counts within reference intervals, i.e. one *A. platys* and *H. canis* co-infected dog with no blood or BM abnormalities and one *H. canis* and three *L. infantum* infected dogs showing BM myeloid and/or megakaryocytic dysplasia, atypical mitosis and suppression of erythroid and/or myeloid lineage.

**Table 2:** Peripheral findings compared with BM pathologic changes (*)

**Blood**							**Bone marrow**				
							**Erythroid suppression, myeloid and or megakaryocyte dysplasia**				
		**Ap**	**Hc**	**Li**	**Co-i**	**Tot**	**Ap**	**Hc**	**Li**	**Co-i**	**Tot**
		**N/10 (%)**	**N/33 (%)**	**N/13 (%)**	**N/27 (%)**	**N/83 (%)**	**N/10 (%)**	**N/33 (%)**	**N/13 (%)**	**N/27 (%)**	**N/83 (%)**
**Normal CBC**		0 (0)	1 (3.0)	3 (23.1)	1 (3.7)	5 (6.0)	0 (0)	1 (3.0)	3 (23.1)^a^	0 (0)^a^	4 (4.8)
**WBC increase in the differential count**		2 (20)	14 (42.4)	1 (7.8)	6 (22.2)	23 (27.7)	2 (20)	12 (36.4)^b^	0 (0)^b^	4 (14.8)	18 (21.7)
**Cytopenia**	Anemia	3 (30)	10 (30.3)	6 (46.2)	15 (55.5)	34 (41.0)	3 (30)	14 (42.4)	7 (53.8)	16 (59.2)	40 (48.2)
	Thrombocytopenia	0 (0)	4 (12.1)	1 (7.8)	1 (3.7)	6 (7.2)					
**Bicytopenia**	Anemia/Thrombocytopenia	1 (10)	4 (12.1)	0 (0)	3 (11.1)	8 (9.6)	2 (20)	4 (12.1)	2 (15.4)	4 (14.8)	12 (14.4)
	Anemia/Neutropenia	1 (10)	0 (0)	2 (15.4)	1 (3.7)	4 (4.8)					
**Pancytopenia**		3 (30)^Cd^	0 (0)^C^	0 (0)	0 (0)^d^	3 (3.6)	3 (30)^Ef^	0 (0)^E^	0 (0)	0 (0)^f^	3 (3.6)
						**Total**	10 (100)	30 (93.9)	12 (92.3)	24(88.92)	77 (92.8)

(*) significant differences are marked by equal letters (lowercase = p < 0.05; uppercase = p < 0.01).

**Table 3:** Main CBC findings in 83 dogs infected by different CVBD pathogens (*)

**CBC**						
		**Ap**	**Hc**	**Li**	**Co-i**	**Tot**
		**N/10 (%)**	**N/33 (%)**	**N/13 (%)**	**N/27 (%)**	**N/83 (%)**
**RBC**	**Normal**	2 (20)	19 (57.6)	5 (38.5)	8 (29.6)	34 (41.0)
	**Anemia**	8 (80)	14 (42.4)^a^	8 (61.5)	19 (70.3)^a^	49 (59.0)
**WBC**	**Normal**	2 (20)	4 (12.1)	6 (46.1)	7 (25.9)	19 (22.9)
	**Leukocytosis**	1 (10)^b^	19 (57.6)^bCd^	1 (7.7)^C^	7 (25.9)^d^	28 (33.7)
	**Leukopenia**	1 (10)	0 (0)	2 (15.4)	0 (0)	3 (3.6)
	**Neutrophilia**	1 (10)	10 (30.3)	1 (7.7)	9 (33.3)	21 (25.3)
	**Neutropenia**	4 (40)^Eg^	0 (0)^E^	2 (15.4)	1 (3.7)^g^	7 (8.4)
	**Eosinophilia**	3 (30)^H^	26 (78.8)^HI^	3 (23.0)^I^	14 (51.8)	46 (55.4)
	**Monocytosis**	1 (10)	5 (15.2)	1 (7.7)	5 (18.5)	12 (14.4)
	**Monocytopenia**	0 (0)	1 (3.0)	0 (0)	0 (0)	1 (1.2)
	**Lymphocytosis**	2 (20)^l^	21 (75.8)^l^	5 (38.5)	13 (48.1)	41 (49.4)
	**Lymphopenia**	1 (10)	0 (0)	1 (7.7)	1 (3.7)	3 (3.6)
**PLT**	**Normal**	6 (60)	25 (75.8)	12 (92.3)	26 (96.3)	69 (83.1)
	**Reduced**	4 (40)^m^	8 (24.2)	1 (7.7)	1 (3.7)^m^	14 (16.9)

(*) significant differences are marked by equal letters (lowercase = p < 0.05; uppercase = p < 0.01).

**Table 4:** BM differential count and dysplastic changes in 83 dogs infected by different CVBD pathogens (*)

**Bone marrow**														
			**Differential count**							**Dysplasia**				
			**Ap**	**Hc**	**Li**	**Co-i**	**Tot**			**Ap**	**Hc**	**Li**	**Co-i**	**Tot**
**BM lineage**			**N/10 (%)**	**N/33 (%)**	**N/13 (%)**	**N/27 (%)**	**N/83 (%)**	**Precursors**	**Abnormalities**	**N/10 (%)**	**N/33 (%)**	**N/13 (%)**	**N/27 (%)**	**N/83 (%)**
**Erythroid**	Normal		6 (60)	20 (60.6)	3 (23.0)	14 (51.8)	43 (51.8)			-				
	Hyperplasia		1 (10)	9 (27.3)	2 (15.4)	7 (25.9)	19 (22.9)	**Promyelocytes**	Bilobated nuclei	-	-	1 (7.7)	1 (3.7)	2 (2.4)
	Hypoplasia		3 (30)	4 (12.1)^A^	8 (61.5)^Ab^	6 (22.2)^b^	21 (25.3)	**Metamyelocytes**	Giants	2 (20)	10 (30.3)	3 (23.0)	6 (22.2)	21 (25.3)
	EMI	Normal	1 (10)^c^	15 (45.4)	2 (15.4)	14 (51.8)^c^	32 (38.5)		Basophilia cytoplasm	-	4 (12.1)	4 (30.8)	4 (14.8)	12 (14.4)
		Increased	9 (90)^D^	18 (54.5)	9 (69.2)	10 (37)^D^	46 (55.4)		Vacuolized cytoplasm	-	-	1 (7.7)	1 (3.7)	2 (2.4)
		Reduced	-	-	2 (15.4)	3 (11.1)	5 (6)	**Banda**	Ring nuclei	-	2 (6)	1 (7.7)	-	3 (3.6)
**Myeloid**	Normal		5 (50)	20 (60.6)	6 (46.1)	14 (51.8)	45 (54.2)		Basophilia cytoplasm	-	1 (3.0)	-	-	1 (1.2)
	Hyperplasia		3 (30)	2 (6)	2 (15.4)	3 (11.1)	10 (12)	**Segmented**	Dohle bodies	3 (30)	6 (18.1)	2 (15.4)	4 (14.8)	15 (18)
	Hypoplasia		2 (20)	11 (33.3)	5 (38.4)	10 (37)	27 (32.5)		Hypersegmented	6 (60)	11 (33.3)	7 (53.8)	10 (37)	34 (41)
	MMI	Normal	2 (20)	16 (48.5)	8 (61.5)	12 (44.4)	38 (45.8)		Basophilia cytoplasm	1 (10)	-	1 (7.7)	-	2 (2.4)
		Increased	3 (30)	6 (18.1)	4 (30.8)	8 (29.6)	21 (25.3)	**Monocytes/ Macrophages**	Vacuolized cytoplasm	3 (30)^O^	0(0)^Op^	1 (7.7)	5 (18.5)^p^	9 (10.8)
		Reduced	5 (50)	11 (33.3)	1 (7.7)	7 (25.9)	24 (28.9)		Erythrophagocytosis	2 (20)	4 (12.1)	-	5 (18.5)	11 (13.2)
	Lymphocytic hyperplasia		2 (20)^e^	10 (30.3)^F^	10 (76.9)^eF^	15 (55.5)	37 (44.6)		Platelet phagocytosis	2 (20)	-	-	1 (3.7)	3 (3.6)
	Plasma cells hyperplasia		2 (20)^g^	15 (45.4)	9 (69.2)^g^	13 (48.1)	39 (47)	**Megakaryocytes**	Dwarfs	4 (40)	8 (24.2)	2 (15.4)	4 (14.8)	18 (21.7)
	Monocytic hyperplasia		9 (90)^h^	15 (45.4)^h^	8 (61.5)	17 (63)	49 (59)		Hyperlobulated nuclei	3 (30)	4 (12.1)	2 (15.4)	2 (7.4)	11 (13.2)
**Megakaryocytes**		Normal	2 (20)	10 (30.3)	4 (30.8)	7 (25.9)	23 (27.7)		Hypolobulated nuclei	-	6 (18.1)	-	1 (3.7)	7 (8.4)
		Hyperplasia	7 (70)	22 (66.6)	6 (46.1)	14 (51.8)	49 (59.0)		Disorganized nuclei	7 (70)^Qrs^	7 (21.2)^Q^	3 (23.0)^r^	6 (22.2)^s^	23 (27.7)
		Hypoplasia	1 (10)	1 (3.0)^i^	3 (23.0)	6 (22.2)^i^	11 (13.2)		Fragmented cytoplasm	6 (60)^TUv^	2 (6)^T^	0(0)^U^	4 (14.8)^v^	12 (14.4)
**Mitosis**		Normal	6 (60)^l^	17 (51.5)^m^	2 (15.3)^lm^	10 (37)	35 (42.1)		Inclusions	2 (20)	-	-	-	2 (2.4)
		Increased	3 (30)	7 (21.2)^n^	8 (61.5)^n^	7 (25.9)	25 (30.1)		Emperipolesis	5 (50)^z^	4 (12.1)^zW^	2 (15.4)	12 (44.4)^W^	33 (39.8)
		Reduced	1 (10)	9 (27.3)	3 (23.0)	10 (37)	23 (27.7)	**Mitosis**	Atypical	0(0)^X^	2 (6)^Y^	(61.5)^XYβ^	3 (11.1) ^β^	10 (12)

(*) significant differences are marked by equal letters (lowercase = p < 0.05; uppercase = p < 0.01).

Eighteen of 23 non-cytopenic dogs (27.7%) with white blood cells count abnormalities including leukocytosis (n = 12/23), neutrophilia (n = 6/23), eosinophilia (n = 20/23), lymphocytosis (n = 15/23) and monocytosis (n = 7/23) were characterized by dysplasia of the myeloid and/or megakaryocytic lineages. Four out of the 23 non-cytopenic dogs had myeloid ‘suppression’ and/or ‘depletion’ without evidence of dysplasia.

Single and multiple cytopenia (Table 2) were identified in 55 out of the 83 dogs enrolled in this study. Forty dogs (48.2%) had a single peripheral cytopenia, of which 34 were anemic and 6 were thrombocytopenic. Three dogs had depletion of all BM lineages in association with severe lymphocytic hyperplasia; seven dogs displayed suppression of erythroid or myeloid lineage, whereas dysplastic changes in one or more BM lineages were detected in the remaining 30 dogs. Twelve out of 83 dogs had peripheral bi-cytopenia, in association with BM differential cell count abnormalities and typical features of dysplasia. Lastly, three pancytopenic *A. platys*-infected dogs, had BM changes consistent with both dysplastic features and abnormalities in the differential count.

## Discussion

In this study, dysmyelopoiesis characterized by the presence of BM dysgranulopoiesis and dysmegakaryocytopoiesis was observed in dogs infected by *L. infantum*, *A. platys* and *H. canis*. Secondary dysmyelopoiesis, which is a non-clonal condition, has generally been associated with the administration of drugs such as estrogen and chloramphenicol, toxin exposure and other specific disease processes, including lead toxicity and iron deficiency [20].

Interestingly, in several instances, BM observations were not in accordance with the corresponding cell counts in peripheral blood, i.e. anemia *vs* erythroid hypoplasia, normal WBC and PLT count *vs* marrow lymphocytosis, monocytosis and megakaryocytic hyperplasia.

In addition, co-infection by two or more CVBD pathogens was associated with more severe BM disorders that were notably different from the abnormalities in dogs infected by a single pathogen.

Normocytic normochromic non-regenerative anemia (NNNA), which was observed in this study (data not shown), is a common finding in CVBDs, including leishmaniasis [1,22], hepatozonoosis [23,24] and, to a lesser extent, anaplasmosis [25]. Interestingly, our data indicates an association between the erythroid hypoplasia and *L. infantum* infection (group Li) when compared to co-infected dogs (Group Co-i) and *H. canis*-infected dogs. NNNA and erythroid hypoplasia with an increase in EMI was associated to small numbers of *L. infantum* parasites in BM samples, in contrast to a previous report in which non-regenerative anemia had been associated with large parasite numbers in the BM [9]. A non-regenerative anemia was also observed in dogs infected by *A. platys* and *H. canis* (group Ap and Hc, respectively) and was associated with an increased EMI, in spite of a reduced or even absent proliferation of erythroid precursors (i.e. rubriblasts and prorubrocytes) in the BM of infected animals. This may be due to the elicitation of a humoral and/or cell-mediated immune response directed against early erythroid precursors [19]. However, further studies are necessary in order to elucidate the relationship/s between infections by *A. platys* and *H. canis* infection and suppression of erythropoiesis.

**Figure 2:**
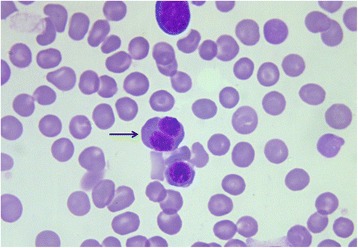
**A megaloblastic erythroid precursor with multiple nuclei (arrow); two other eryhtroid precursors are also present.** Bone marrow. Quick Stain, 100×.

Erythroid hyperplasia and mild dyserythropoiesis characterized by megaloblastic erythroid cells with multiple nuclei (Figure 2) were observed in two dogs <8 months of age. These morphologic abnormalities are most often seen in animals with myeloproliferative disorders, congenital dyserythropoiesis or following treatment with chemotherapeutic drugs [26]. It was unclear whether the changes observed in the BM of the two young animals were associated to an evolving myelodysplastic syndrome not yet manifested.

Eosinophilia was observed in 55.4% of the dogs and more frequently in *H. canis*-infected dogs. Examination of BM smears from these dogs revealed myeloid cells within reference intervals or myeloid hypoplasia in association with an increased BM eosinophilic component (data not shown). Peripheral eosinophilia has already been linked to *H. canis* [24,27] and canine *Bartonella* infections [28,29]; however, it is important to note that this condition may also be caused by a number of diseases other than CVBDs [30], as well as by concomitant infections by gastrointestinal parasites which were not investigated in dogs included in this study. Leukocytosis was often observed in *H. canis* infected dogs (group Hc); interestingly, neutrophilia, which is usually associated with high rates of parasitemia [24], was not recorded in this study, possibly as a consequence of the small numbers of parasitized neutrophils (data not shown).

Myeloid hypoplasia, in association with normal peripheral WBC counts or with neutrophilia was observed in 11 (33.3%) *H. canis*-infected dogs and in 10 (37%) *H. canis - L. infantum* co-infected dogs. This finding is not easily explained as granulocytic hypoplasia is generally associated with peripheral neutropenia. The BM of these animals was considered suppressed due to an absence of proliferation (MMI increased). It is possible that the mature neutrophilia in peripheral blood with simultaneous suppression of the myelocytic cell lines may be due to other factors such as mobilization and demargination of neutrophils due to stress and excitement [31].

**Figure 3:**
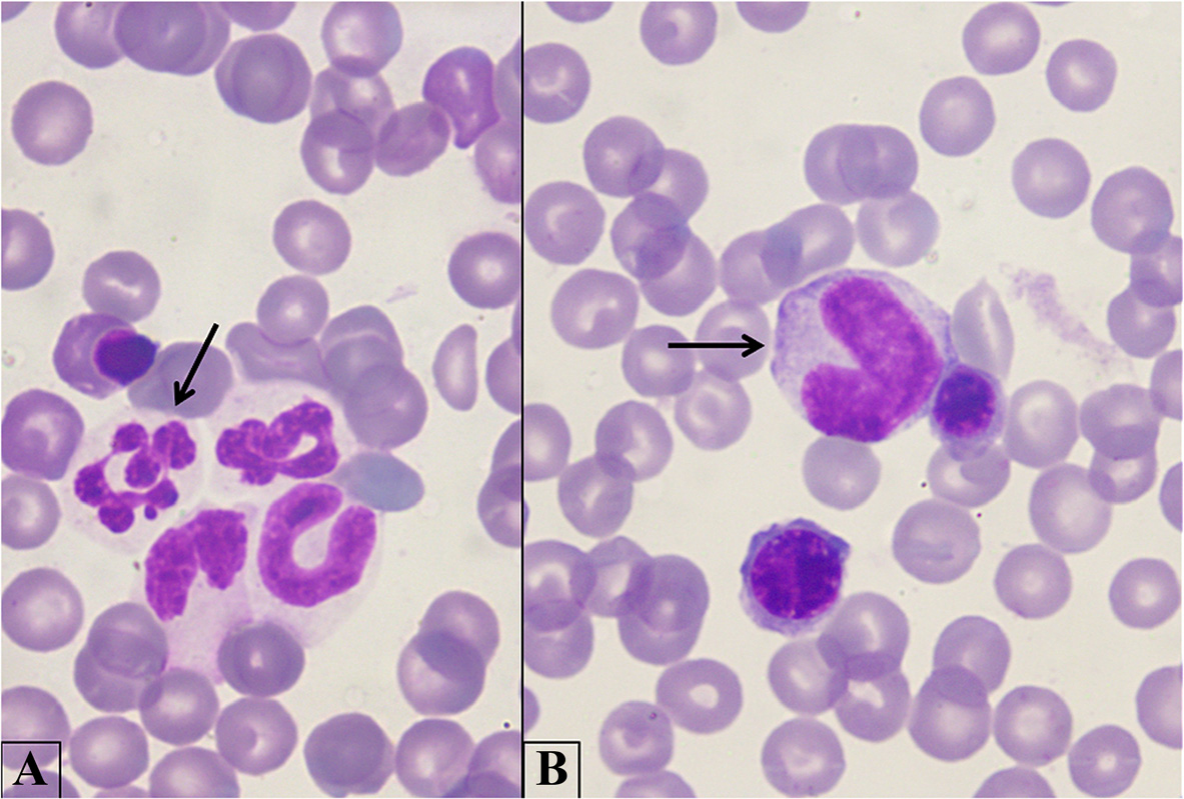
**Myeloid dysplasia. A**. A hypersegmented mature neutrophil (arrow); myeloid precursors in different maturation stages are also present. **B**. A giant metamyelocyte (arrow) with two erythroid precursors. Bone marrow. Quick Stain, 100×.

Dysmorphic changes in neutrophilic granulocytes, which generally occurs in animals with inflammatory diseases and/or dysgranulopoiesis [32,33] were observed, with varying prevalence (Table 4, Figures 3B, 1). Therefore, vector-borne pathogens, and in particular *H. canis*, should be considered among the secondary causes of dysgranulopoiesis in dogs. Indeed, morphological abnormalities were also observed in peripheral neutrophils (data not shown, Figure 3A) of dogs infected by *H. canis* (group Hc) and in *H. canis* co-infected dogs (Group Co-i).

Severe BM lymphocytosis, plasma cell hyperplasia and/or monocytosis were frequently observed in dogs infected with *A. platys*, *L. infantum* and *H. canis*. This may be related to chronic antigenic stimulation as it has been shown for other CVBDs, such as canine monocytic ehrlichiosis [34]. Interestingly, these BM changes were not reflected in CBCs, since most dogs had peripheral blood lymphocytes and monocytes within reference intervals.

**Figure 4:**
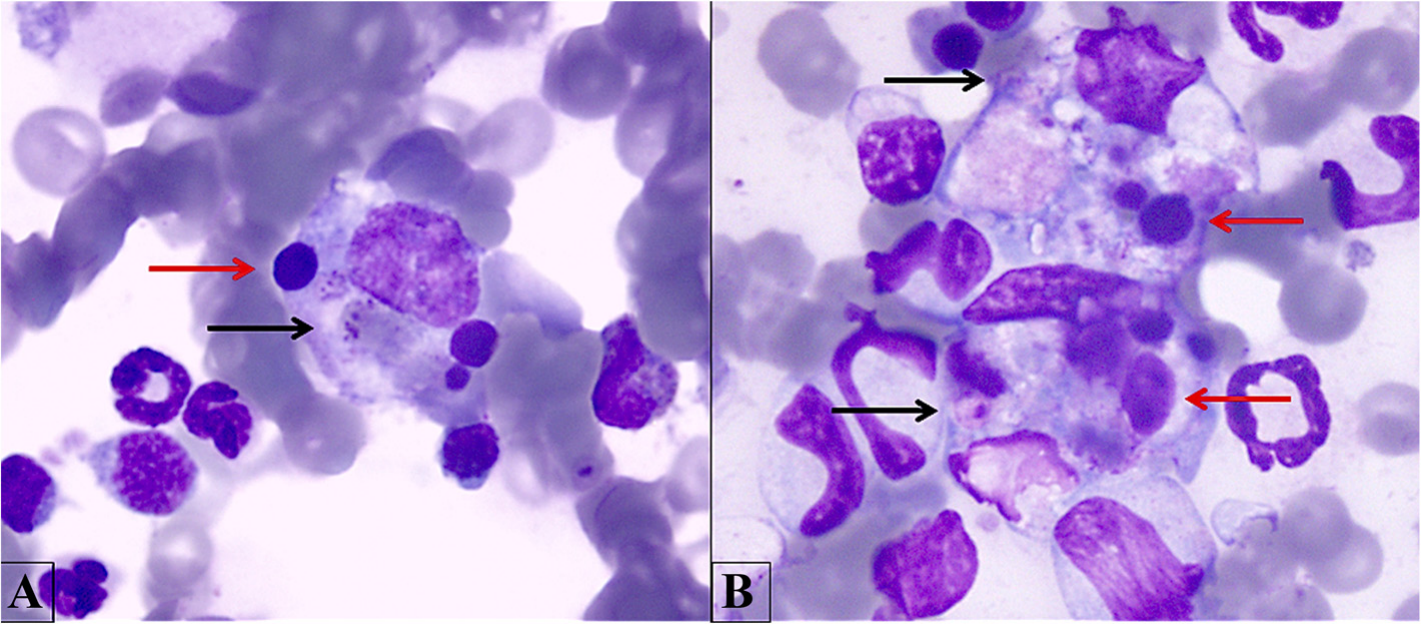
**Platelets and erythrophagocytosis. A**. A macrophage phagocyting erythroid precursors (red arrow), platelets (black arrow) and cellular debris. **B**. Two macrophages with the same features described in A. Bone marrow. Quick Stain, 100×.

Hemophagocytic macrophages containing erythrocytes were observed in the BM of *A. platys* and *H. canis* infected dogs, whereas these cells were not detected in BM samples from *L. infantum* infected dogs, in contrast to a previous report [8]. Erythophagocytosis may be immune-mediated, due to antibody or possibly complement deposition on the surface of red cells, as it has been described for several vector-borne diseases [8]. It is also possible that infection-induced red cell abnormalities may alter membrane properties that result in reduced red cell lifespan and trigger phagocytosis [33]. Erythrophagocytosis may also occur as an “innocent bystander effect” whereby phagocytic macrophages remove red cells in the process of removing cellular debris, such as in the case of disseminated fungal infection [35], lymphocytic leukemia, myelomonocytic leukemia [36] and BM necrosis [37]. Erythrophagocytosis and platelet phagocytosis were observed in four *A. platys* infected dogs (Figure 4A,B), consistent with the immune-mediated thrombocytopenia that this pathogen is known to induce [38,39].

**Figure 5:**
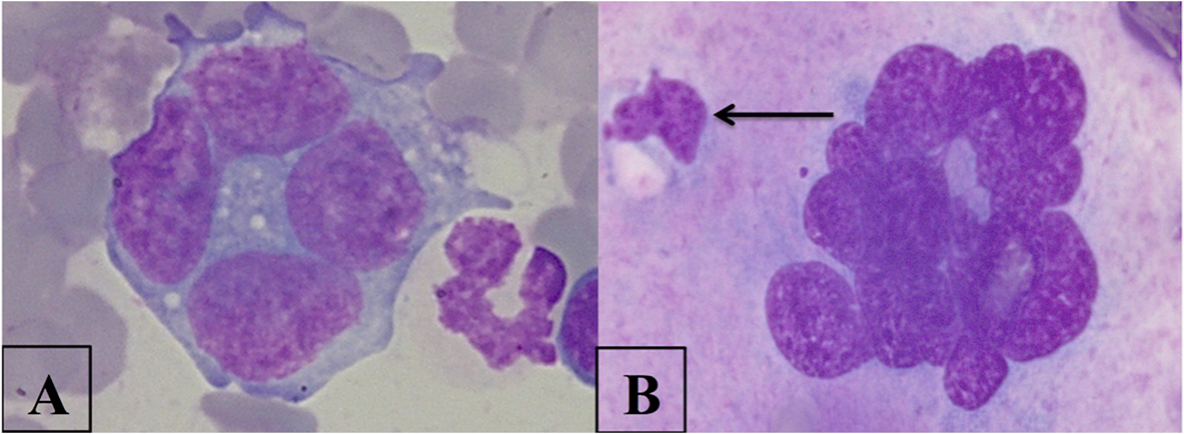
**Megakaryocytic dysplasia. A**. Megakaryocytic precursor with four divided nuclei. **B**. A megakaryocyte presenting morphologic abnormalities in nucleus shape and emperipolesis (arrow). Bone marrow. Quick Stain, 100×.

Megakaryocytic hyperplasia and dysplasia were the most frequent BM findings in this study. They were observed in all CVBD infection groups and they were especially frequent in *A. platys* infected dogs. Despite the presence of these abnormalities in the BM, numbers of platelets in peripheral blood were frequently normal. Features of megakaryocytic dysplasia included asynchronous maturation resulting in dwarf granular megakaryocytes, hyperlobulated, hypolobulated and/or disorganized nuclei (Figure 5A,B) and cytoplasmic abnormalities (tattered fringe and inclusions). We hypothesize an immune-mediated cause for the megakaryocytic dysplasia observed, in accordance with previous reports of dogs infected by *L. infantum* [8].

**Figure 6:**
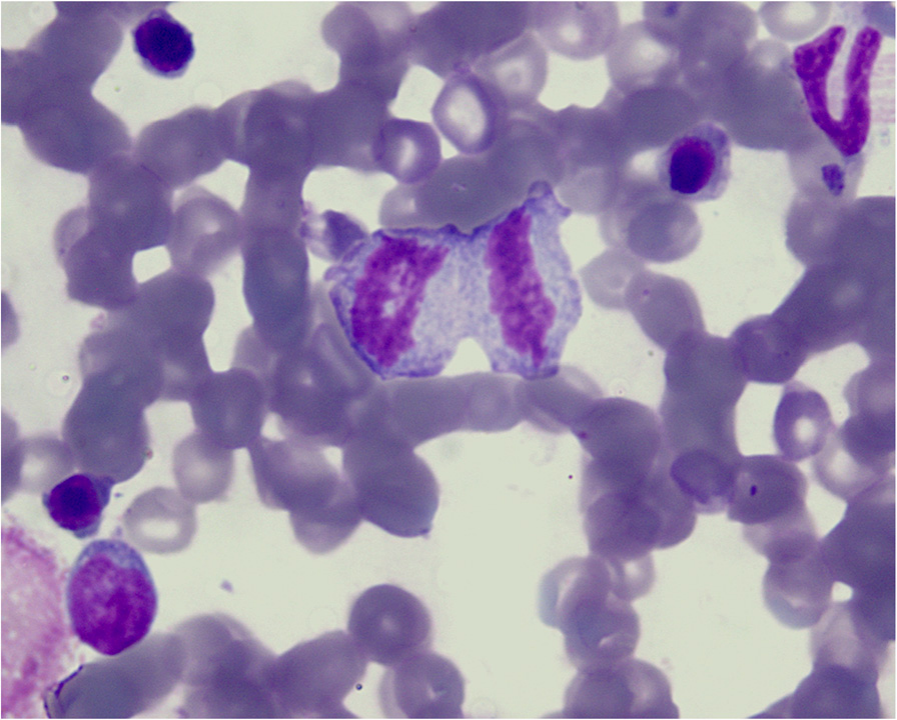
**Atypical mitosis.** Bone marrow. Quick Stain, 100×.

Emperipolesis (Figure 5B), i.e. engulfing of various BM cells by megakaryocytes [40], was also frequently observed in this study. Emperipolesis increases in various disorders, including multiple myeloma, carcinoma, lymphoma [41], platelet disorders [42] and serious hemorrhage [43], however the pathophysiological importance of emperipolesisis is yet to be fully understood and its significance is uncertain [44,45]. Indeed, this event may be linked to the effects of interleukin-6 (IL-6), which alters the process of megakaryocyte maturation, thus altering thrombocytopoiesis [45]. To our knowledge, prior to the present study, emperipolesis had only been described in dogs infected by *L. infantum* [8] and *A. platys* [39]; we describe, for the first time, emperipolesis in dogs infected or co-infected by *H. canis*. Lastly, atypical mitotic figures were noted infrequently in dogs infected or co-infected by *H. canis* and *L. infantum* (Figure 6), thus raising the question of whether selected CVBDs may indeed represent previously unrecognized causes of secondary dysmyelopoiesis.

## Conclusions

This study provides information on the cytological disorders and pathological changes occurring in the BM of dogs naturally infected by a range of CVBD-causing pathogens. Dysgranulopoiesis and dysmegakaryocytopoiesis (i.e. emperipolesis) were typical BM alterations associated with *H. canis* infections. Megakaryocytic hyperplasia and dysplasia were observed in *A. platys* infected dogs, even in absence of thrombocytopenia at the time of testing, whilst infection with *L. infantum* was linked to BM erythroid suppression, infiltration of the BM with lymphocytes and plasma cells and the presence of atypical mitosis. Finally, more severe BM disorders were observed in dogs co-infected by two or more CVBD pathogens than in dogs infected by a single agent. Based on our findings, infections by vector-borne pathogens should be considered as differential diagnoses for secondary myelodysplasia in dogs. Additional studies that further describe and explore mechanisms of BM changes induced by these agents and their relationship with observed changes in peripheral blood cell counts during the course of infection appear warranted.

## Abbreviations

BM: Bone marrow
CVBD: Canine vector-borne disease
M:E: Myeloid to erythroid ratio
EMI: Erythroid maturation index
MMI: Myeloid maturation index
CBC: Complete blood count
PCR: Polymerase chain reaction
WBC: White blood cells
RBC: Red blood cells
PLT: Platelets
NNNA: Normocytic normochromic non-regenerative anemia
